# Ultrasound-Guided Percutaneous Fasciotomies for Patients With Chronic Exertional Compartment Syndrome

**DOI:** 10.1016/j.eats.2024.103119

**Published:** 2024-09-23

**Authors:** Axel Machado, Tristan Fauchille, Rayan Fairag, Jonathan Cornacchini, Nicolas Bronsard, Nicolas Ciais, Jean-François Gonzalez, Alexandre Rudel, Grégoire Micicoi

**Affiliations:** a iULS–University Institute for Locomotion and Sports, Hôpital Pasteur 2, University Côte d'Azur, Nice, France; b Department of Radiology, Centre Hospitalier Universitaire de Nice, Hôpital Pasteur 2, Nice, France

## Abstract

Chronic exertional compartment syndrome is a well-described potential cause of leg pain in high-level athletes and soldiers. Surgical treatment of chronic exertional compartment syndrome usually involves fasciotomy, with a reported rate of complications of up to 16%, including failure of complete compartmental release and delayed return to normal daily activity, which can take up to 6 to 12 weeks. The use of a minimally invasive approach under ultrasound guidance seems to improve clinical outcomes in young active patients. We recommend the following steps for effective execution of ultrasound-guided percutaneous fasciotomy: (1) location of the compartmental fascia and identification of the superficial peroneal nerve, (2) skin incision, (3) insertion of a hook under the compartmental fascia, and (4) sectioning of the fascia.

Chronic exertional compartment syndrome (CECS) is a well-described potential cause of leg pain in high-level athletes and soldiers.^1^ When all medical options fail, surgical treatment involving fasciotomy may be proposed, allowing 81% to 100% symptom relief.^2^ Despite the apparent simplicity of the operation, procedural complications have been reported at a rate of up to 16% and the return to unrestricted activity can take up to 6 to 12 weeks. This complication rate is increased when the skin is also opened with a large approach performed for compartmental decompression.^3^

However, superficial peroneal nerve injuries are one of the main complications during minimally invasive surgery without visual control (endoscopy or ultrasound) of the nerve.^4^ Direct endoscopic visualization provides excellent results, with an overall 79.9% return-to-sports rate. However, the most common complication is postoperative hematoma, and although endoscopic control allows direct visualization of the fascia, it does not allow perfect control of the superficial peroneal nerve.

To avoid the aforementioned morbidity, minimally invasive surgical techniques, such as ultrasound-assisted surgical fasciotomy using long Metzenbaum scissors, have been described, with positive outcomes and no reported complications in a case series of 7 patients with anterior CECS.^5^ A recent case report described the successful treatment of a 41-year-old runner in whom a 3-mm incision was performed with a V-shaped meniscotome in the office while under local anesthesia, allowing the athlete to return to running within 1 week of the procedure without any symptom recurrence at 9-month follow-up.^6^ The use of a minimally invasive approach under ultrasound guidance (USG) seems to improve clinical outcomes in young active patients.

## Surgical Technique

### Equipment

**Fig 1:**
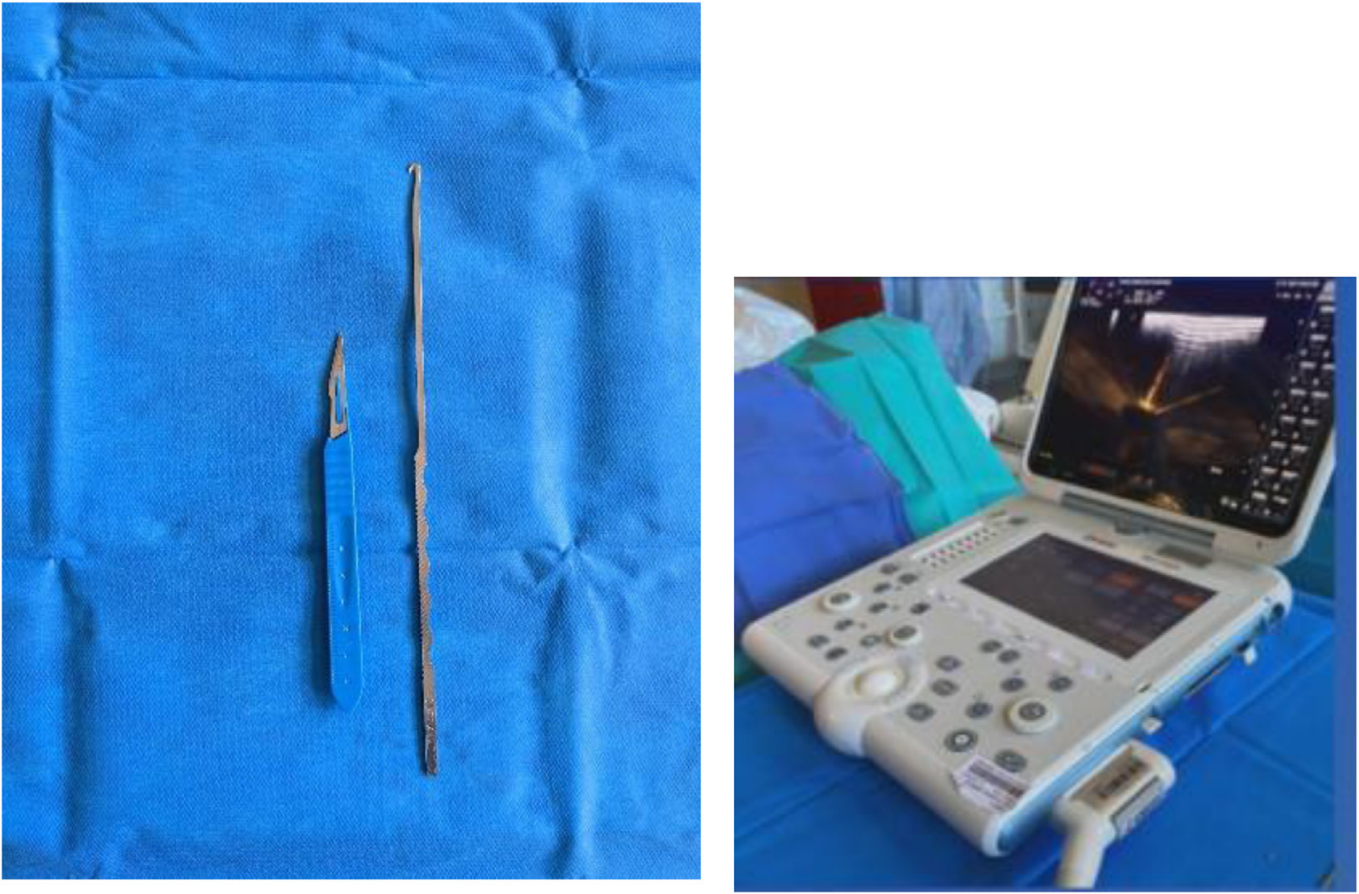
Scalpel, hooked blade, and ultrasound machine with linear 8- to 12-MHz transducer used for fasciotomy.

The only equipment needed is a scalpel and a hooked blade. Our technique is performed under ultrasound control; in the presented case, we used an ultrasound machine with a linear 8- to 12-MHz transducer (Fig 1).

### Ultrasonographic Percutaneous Fasciotomy Technique

**Fig 2:**
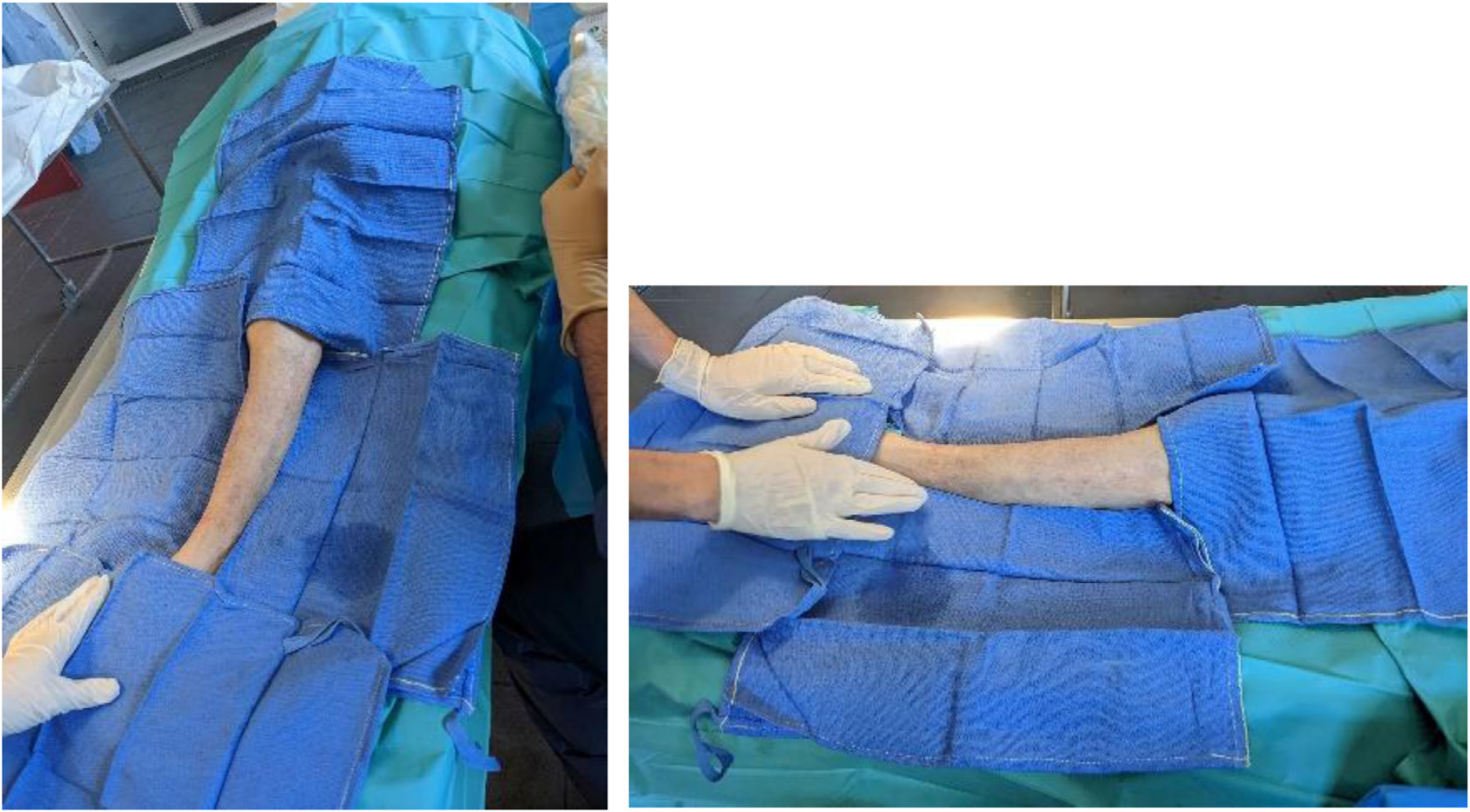
Surgical positioning of a left leg.

The patient is placed in the supine position with the foot in mild internal rotation to expose the anterior and lateral compartments of the leg (Fig 2).

##### Location of Compartmental Fascia and Identification of Superficial Peroneal Nerve (Anterior Compartment)

**Fig 3:**
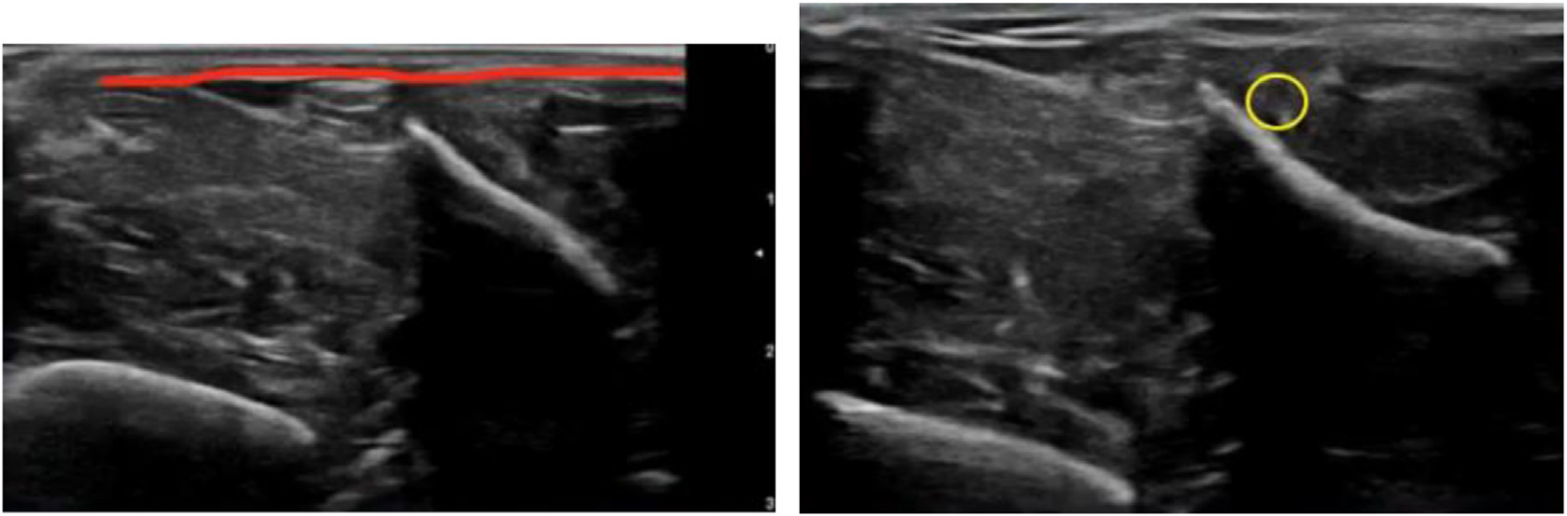
Ultrasound view. The fascia is indicated in red. The yellow circle indicates the superficial fibular nerve.

The first step is to locate the fascia using ultrasound and then to locate the superficial fibular nerve, which must be guarded throughout the procedure to avoid iatrogenic sectioning (Fig 3).

##### Skin Incision

**Fig 4:**
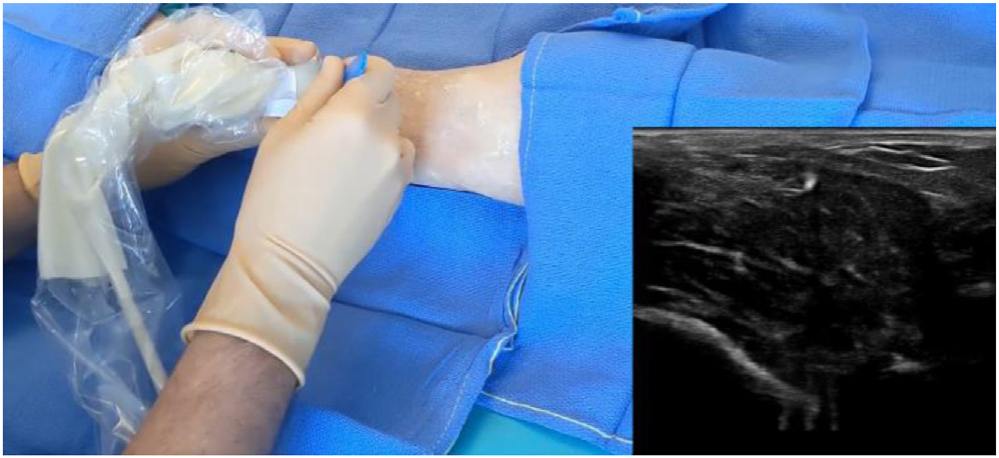
Scalpel blade crossing skin in a right leg, followed by fascia.

Next, a millimeter skin incision centered in the middle of the leg is made under USG (Fig 4).

##### Insertion of Hook Under Compartmental Fascia

**Fig 5:**
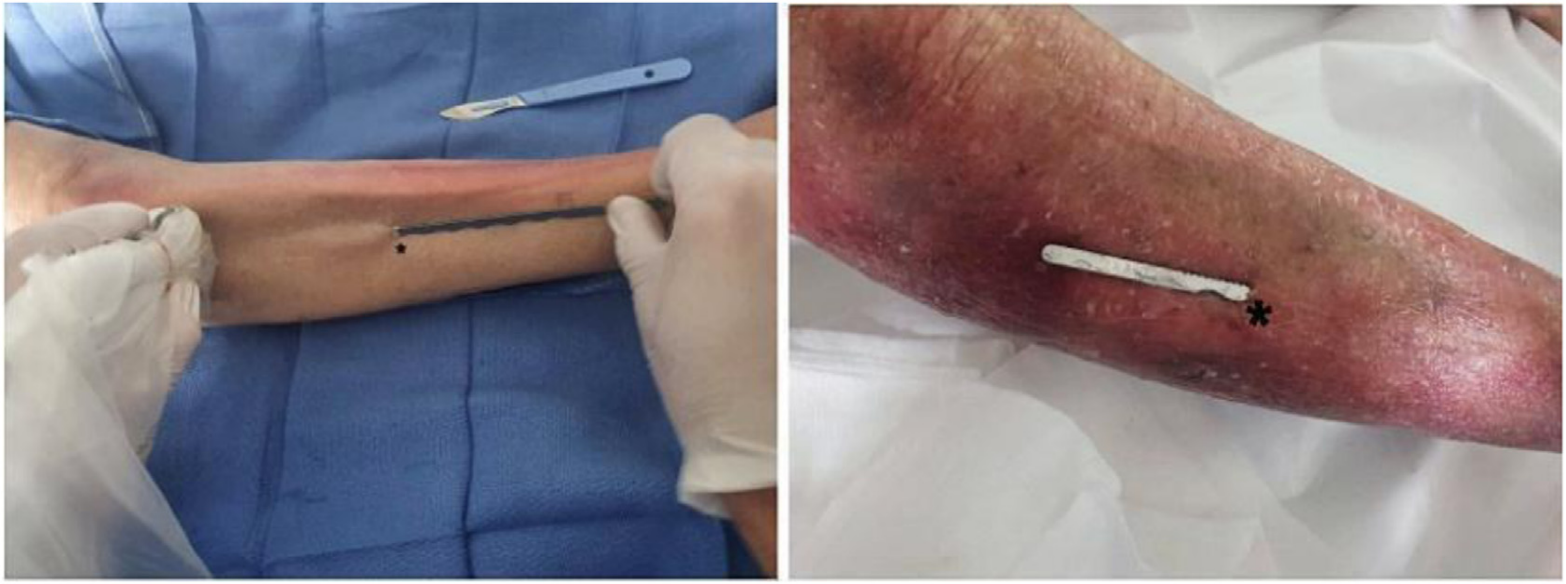
Intraoperative images of right leg showing use of ultrasound guidance while performing proximal division of fascia over anterior compartment. The asterisks indicate the minimally invasive incision.

The hooked blade is introduced under the fascia, and the hook is then slid under the fascia to the distal part of the section. The surgeon must always pay attention to the superficial fibular nerve, which was located and drawn beforehand (Fig 5).

##### Sectioning of Fascia

**Fig 6:**
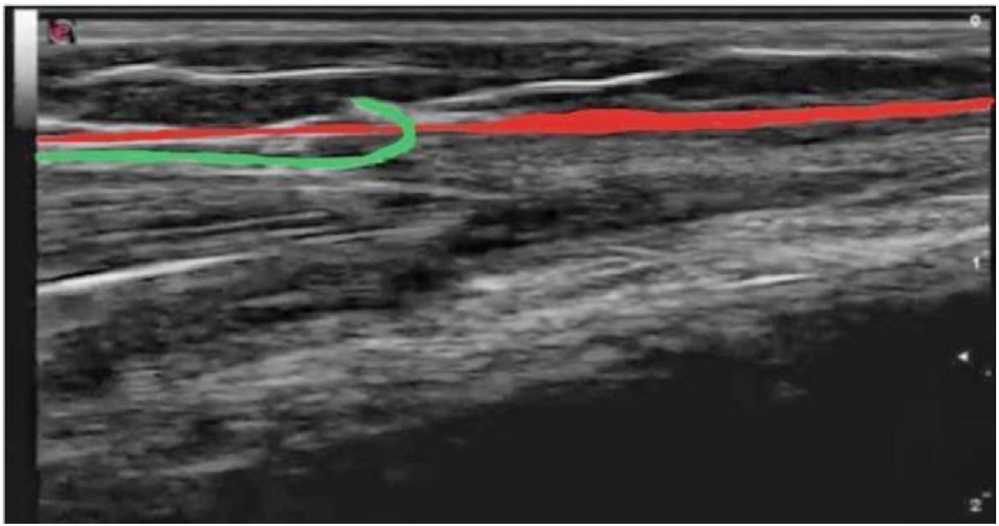
Ultrasound picture of hook dividing fascia (green). The hyperechoic fascia is indicated in red.

**Fig 7:**
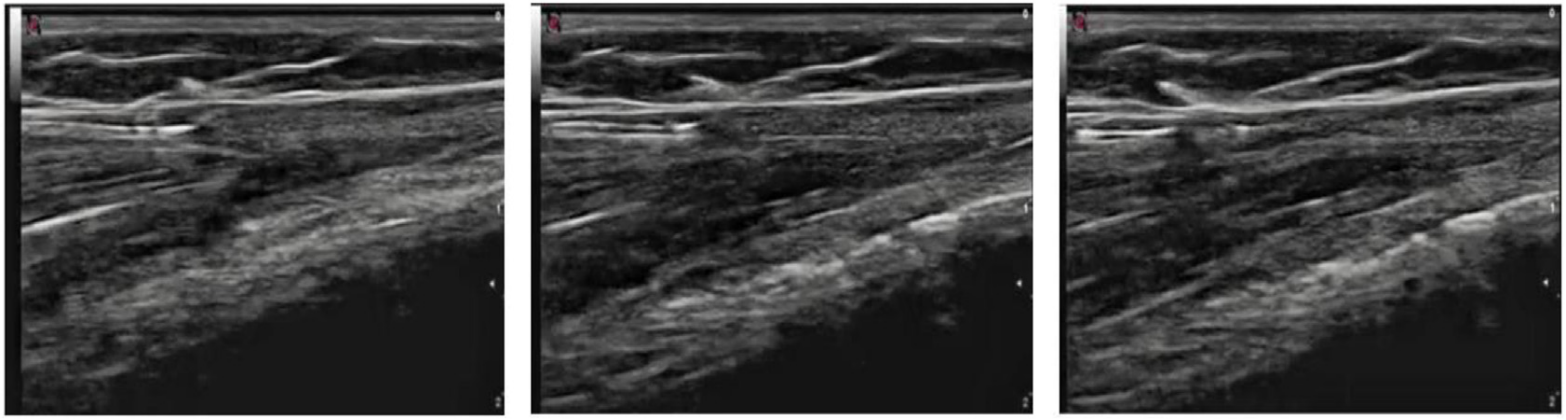
The hook is introduced under the fascia; the fascia is then incised in a retrograde manner.

**Fig 8:**
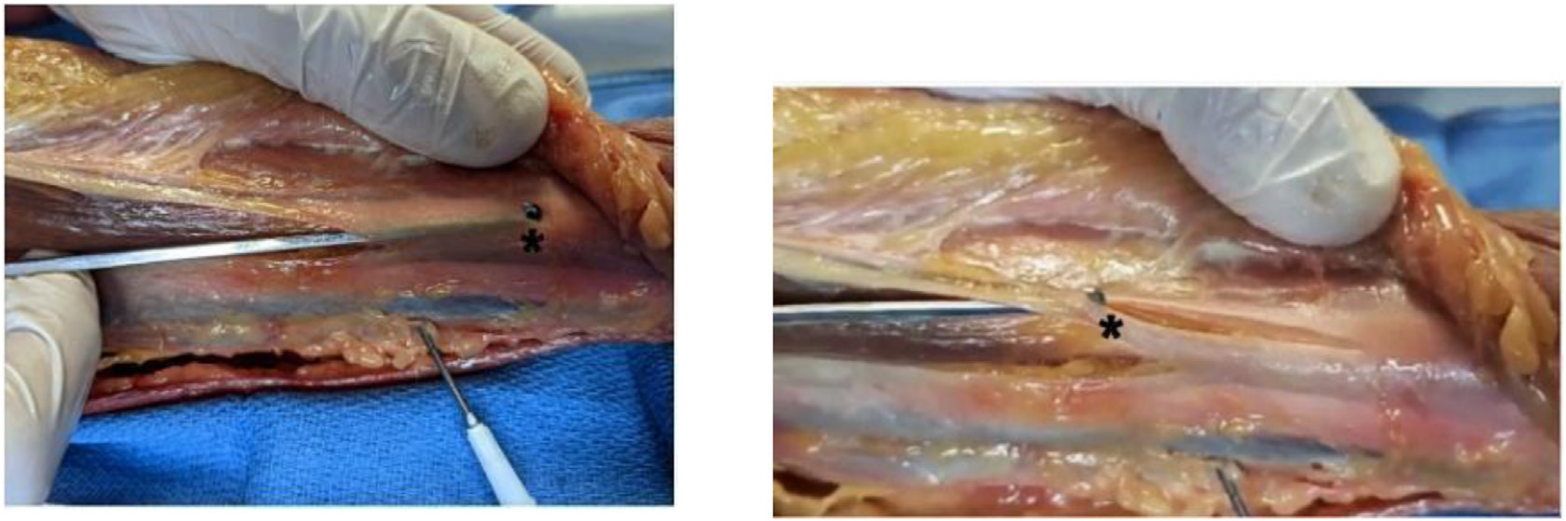
Open view of hook cutting fascia (asterisks) of a right leg.

**Fig 9:**
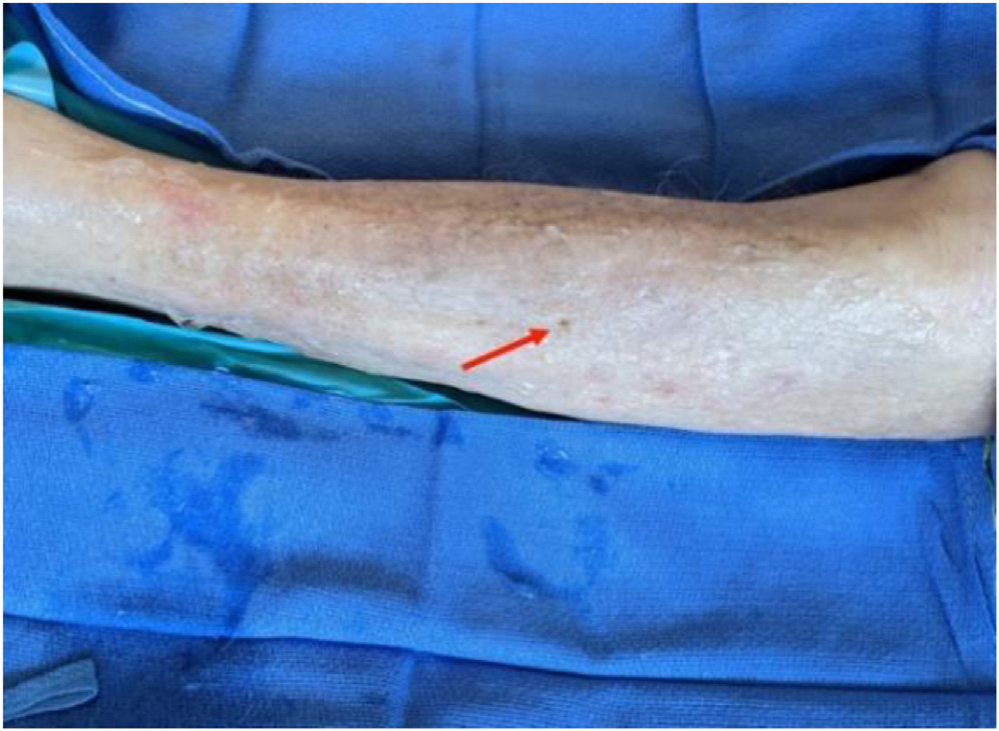
Postoperative aspect of millimeter skin incision (arrow). If it is impossible to cut the entire fascia, 1 or 2 additional millimeter incisions may be necessary.

The hooked blade is turned 90° to incise the fascia and perform sectioning in a retrograde manner by pulling out the hook. The surgeon should take care to avoid sectioning too superficially to prevent damaging the skin (Fig 6, Fig 7, Fig 8). The procedure is repeated proximally from the same incision. The same procedure is then repeated for the lateral compartment with a millimeter skin incision (Fig 9).

### Feasibility and Safety Assessment

**Fig 10:**
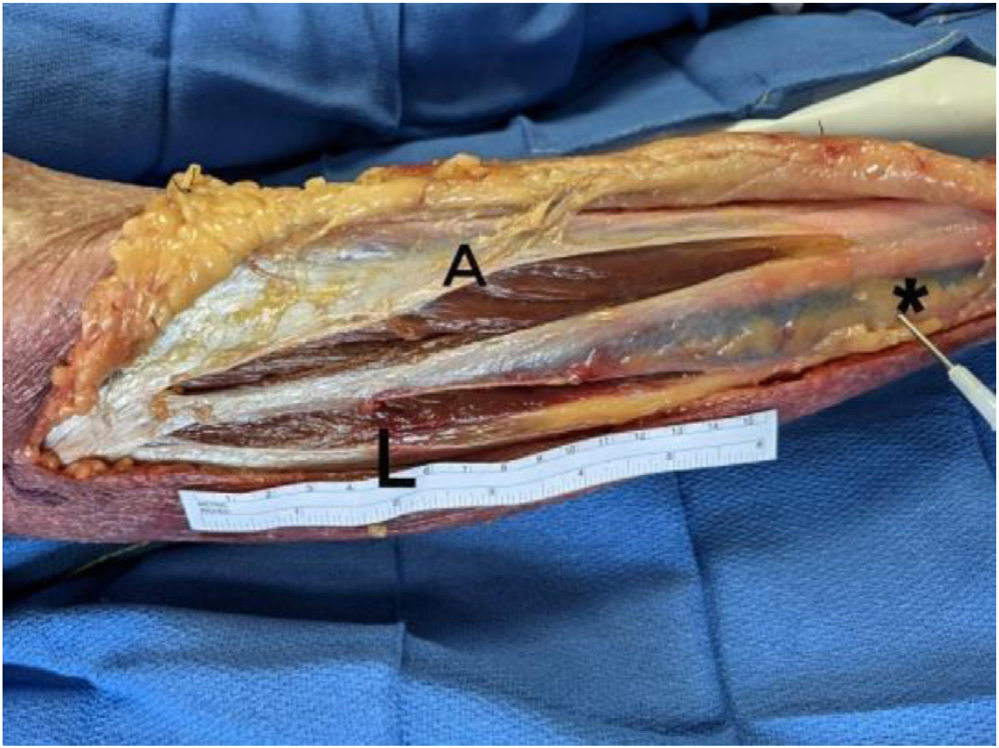
Dissection with measurement of fasciotomy length. The asterisk indicates spotting and marking of the superficial fibular nerve under ultrasound. (A, anterior compartment; L, peroneal compartment.)

The completeness of the fascial release is determined after dissection and direct visualization. The fasciotomy is classified as “complete” if its total length is 10 cm or greater.^7^ The continuity of the fascial transection is described as either continuous (i.e., no intact fascial segments) or discontinuous (i.e., intact fascial segments). The superficial peroneal nerve is dissected distally to verify its integrity (Fig 10). Video 1 shows ultrasound-guided percutaneous fasciotomy in a cadaveric specimen, and Table 1 summarizes the main results of the described intervention in 6 cadaveric specimens.

**Table 1:** Main Results of Intervention in 6 Cadaveric Specimens

Characteristic	Data
Sex, n	3 F and 3 M
Mean age, yr	83.2
Side, n	6 R and 6 L
Mean duration (range), min	13.5 (10-18)
Mean anterior compartment section (range), cm	19.4 (14-26)
Mean lateral compartment section (range), cm	13.2 (2-20)
Successful anterior compartment fasciotomy (>10 cm), % (n)	100 (12 of 12)
Successful lateral compartment fasciotomy (>10 cm), % (n)	83 (10 of 12)
Continuous anterior fasciotomy, % (n)	100 (12 of 12)
Continuous lateral fasciotomy, % (n)	83 (10 of 12)
Nerve injury, % (n)	0 (0 of 12)

F, female; L, left; M, male; R, right.

## Discussion

No tendon, muscle, or vascular-nervous injuries were observed after 24 USG fasciotomies were performed, supporting the notion that this procedure can be achieved in a safe manner. The primary risk during fasciotomy is injury to the nerve branches of the superficial fibular nerve,^4^^,^^8^ but this risk is limited by the identification under USG.

Using the hook by a single few millimeters incision through the fascia enables a minimally invasive approach. By making the incision in the middle third of the leg, the hook can be advanced proximally and distally to complete the fasciotomy without the need for additional assistance, as described in previous studies.^7^ These results support the notion that this technique would be feasible in the office with the patient under local anesthesia; a complementary injection would also allow “hydro-dissection” of the fascial planes and thus facilitate the procedure.

Further studies are necessary to confirm the use of the described technique in clinical practice. Clinical studies should compare the results with standard approaches, and cost analyses should be performed given that this technique could be less expensive if the procedure potentially could be performed directly in the outpatient clinic setting with local anesthesia, thus avoiding the cost of an operating room and surgical materials.

In conclusion, USG fasciotomy is a feasible and safe procedure in a cadaveric model. This approach may allow decreases in morbidity and cost compared with open surgical procedures, but clinical studies will be necessary to confirm the efficiency and efficacy of symptom relief in young active athletes with CECS.

## Declaration of Generative AI and AI-Assisted Technologies in the Writing Process

During the preparation of this work, the authors used Murf AI for the audio commentary in Video 1. After using this tool/service, the authors reviewed and edited the content as needed and take full responsibility for the content of the publication.

## Disclosures

All authors (A.M., R.F., J.C., N.B., N.C., J-F.G., A.R., G.M.) declare that they have no known competing financial interests or personal relationships that could have appeared to influence the work reported in this paper.

## Supplementary Data

Video 1 Ultrasound-guided percutaneous fasciotomy in cadaveric specimen.
